# Occurrence of scorpion sting and associated factors in a highly marginalized municipality in Guerrero, Mexico: A cross-sectional study

**DOI:** 10.1371/journal.pntd.0011271

**Published:** 2023-05-01

**Authors:** Blanca Estela Trinidad-Porfirio, Arcadio Morales-Pérez, Elizabeth Nava-Aguilera, Miguel Flores-Moreno, Liliana Morales-Nava, Jaime García-Leyva, Rufino Silva-Domínguez, Antonio Juan Cortés-Guzmán, Ildefonso Fernández-Salas, Neil Andersson

**Affiliations:** 1 Centro de Investigación de Enfermedades Tropicales, Universidad Autónoma de Guerrero, Acapulco, Guerrero, México; 2 Departamento de Epidemiología y Medicina Preventiva, Secretaría de Salud Guerrero, Chilpancingo, Guerrero, México; 3 Laboratorio de Entomología Médica, Facultad de Ciencias Biológicas, Universidad Autónoma de Nuevo León, San Nicolás de los Garza, Nuevo León, México; 4 Department of Family Medicine, McGill University, Montreal, Canada; Fundação de Medicina Tropical Doutor Heitor Vieira Dourado, BRAZIL

## Abstract

**Background:**

Scorpion sting is a neglected public health problem, despite a global estimate of 1.2 million scorpion stings and some 3,250 deaths annually

**Methods:**

This cross-sectional study estimates the occurrence of scorpion stings and identifies associated factors in seven communities in the highly marginalized municipality of Chilapa, in the Mexican state of Guerrero. After informed consent, 1,144 households provided information on 4,985 residents. The questionnaire collated sociodemographic data, characteristics of the dwelling, efforts to avoid scorpion stings, and individual information of scorpion stings suffered in the last year. Cluster-adjusted (acl), bivariate and multivariate analysis relied on the Mantel-Haenszel procedure

**Results:**

The overall period prevalence of scorpion stings in the year prior to the study was 4.4% (218/4985), 5.4% in men (126/2320), and 3.5% in women (92/2665), p<0.01. The majority occurred at home 68.3% (149/218), followed by agricultural fields 26.6% (58/218), street 2.8% (6/218), and work 2.3% (5/218). Factors associated with scorpion sting were carrying firewood (OR 2.1; CI95%acl 1.40–3.09), keeping free-range hens around of the home (OR 1.9; CI95%acl 1.19–2.85), residing in a rural area (OR 1.7; CI95%acl 1.04–2.78), being male (OR 1.6; CI95%acl 1.18–2.28), and helping with housework (OR 1.6; CI95%acl 1.04–2.40)

**Conclusion:**

This study confirms scorpion bites are a public health problem in these marginalized communities in Guerrero State, with risk factors related to living conditions and the work process at home and in the fields. Almost all risk factors identified could be reduced with low-cost interventions implemented by the communities themselves.

## Author summary

A high incidence of scorpion sting affects several Mexican states, including Guerrero, sometimes resulting in death. This cross-sectional study in seven highly marginalized communities of Chilapa Municipality administered a household survey to collate information on 4,898 people. Analysis focused on scorpion stings and risk factors related to the work processes of women and men, including cooking and housework. Men were at greater risk due to their agricultural work. Other factors like keeping poultry and storing firewood in the home also carry higher risks. Targeted low cost local actions could reduce the risks substantially.

## Introduction

Scorpionism is a public health problem, with more than 1200000 stings and 3250 deaths registered each year in the world, an incidence of 526 per 100,000 people and mortality rate of 0.27% [1]. Mexico reports high rates of scorpion stings [2,3], an overall incidence of 233.64 per 100000 [1]. Several states report much higher levels. Colima, for example, reported 1423.6 per 100000 people, Nayarit 1392.7, Morelos 1367.7, and Guerrero 1137.4. Chilapa municipality in Guerrero reports the highest incidence in the state, 1434 per 100000 inhabitants [4].

Depending on the species, scorpion stings cause different degrees of envenomation. Mild symptoms include pain at the site of the sting and local paresthesia, while moderate symptoms can range from sialorrhea, general paresthesia, abdominal distension, fasciculations, dyspnea, and retrosternal pain. Severe symptoms include dysphagia, nystagmus, convulsions, vomiting, ataxic gait, transient blindness, high blood pressure, priapism, vaginal irritation, and acute pulmonary edema. A small proportion of stings cause organ damage and can lead to death [5].

Of 289 scorpion species reported in Mexico, 19 are of medical significance [6] and seven of these infest Guerrero State [7,8]. Chilapa municipal records show at least two species: *Centruroides villegasi and Centruroides limpidus* [8,9].

Epidemiological studies suggest a majority of scorpion stings occur at home [10,11], mainly at night due to the nocturnal habits of arachnids [10,11,12]. Some authors report higher risks in rural populations [11,13], while others suggest urban populations are worse affected [14,15]. Risk factors for scorpion sting include being in a rural area [14]; insufficient household cleaning; roofing constructed from temporary sheeting or palm leaves; keeping ducks in the home; agricultural work [16]; an altitude of 300 meters above sea level; being male; being mixed race (as opposed to indigenous); and, not using gloves when harvesting corn [17].

Prevention and control measures for scorpion stings include individual measures such as shaking out bedclothes, clothing, and footwear before use and, for agricultural workers, the use of adequate protective clothing and equipment [18,19]. Household measures include cleaning and dusting walls and furniture; installing a ceiling in bedrooms; installing fly screens and mosquito nets; painting bed legs; removing household construction material and trash; cleaning and weeding the area around the home; and, plastering and painting walls. At the community level, measures include informing the local population via workshops [20]. The present study aimed to estimate scorpion stings’ occurrence and identify the associated risk factors.

## Material and methods

### Ethics statement

The Ethics Committee of the Centro de Investigación de Enfermedades Tropicales (CIET) approved the study in May 7, 2018. After receiving an explanation of the objectives and procedures of the study, the community authorities gave their permission for the study. Before each interview, the interviewer explained the objectives of the research and that participation was voluntary, confidential, and anonymous. They explained the right to refuse to participate or not to respond to any question, and that they could stop the interview at any point, a decision that would be respected without affecting the care they receive from local health services. Oral consent was obtained because many people do not know how to write and distrust signing documents but trust word of mouth. Oral consent was recorded in the survey notebooks at the time households gave access to their homes.

### Study setting and sampling

The southern state of Guerrero (population 3.5m) is one of the poorest in Mexico and has a large indigenous population. Chilapa has 123,722 inhabitants, 40% of whom live in urban areas [21]. Some 65% of households have no access to running water and 63% cook with firewood or coal [22]. Chilapa municipality is in the central region of Guerrero. It is located between the coordinates of north latitude 17°35’39", west longitude 99°10’40" and altitude of 1406 meters above sea level. The climate is predominantly semi-warm sub-humid and average annual temperature of 20°C [23] with rainfall of 800 to 2000 millimeters [24].

**Fig 1:**
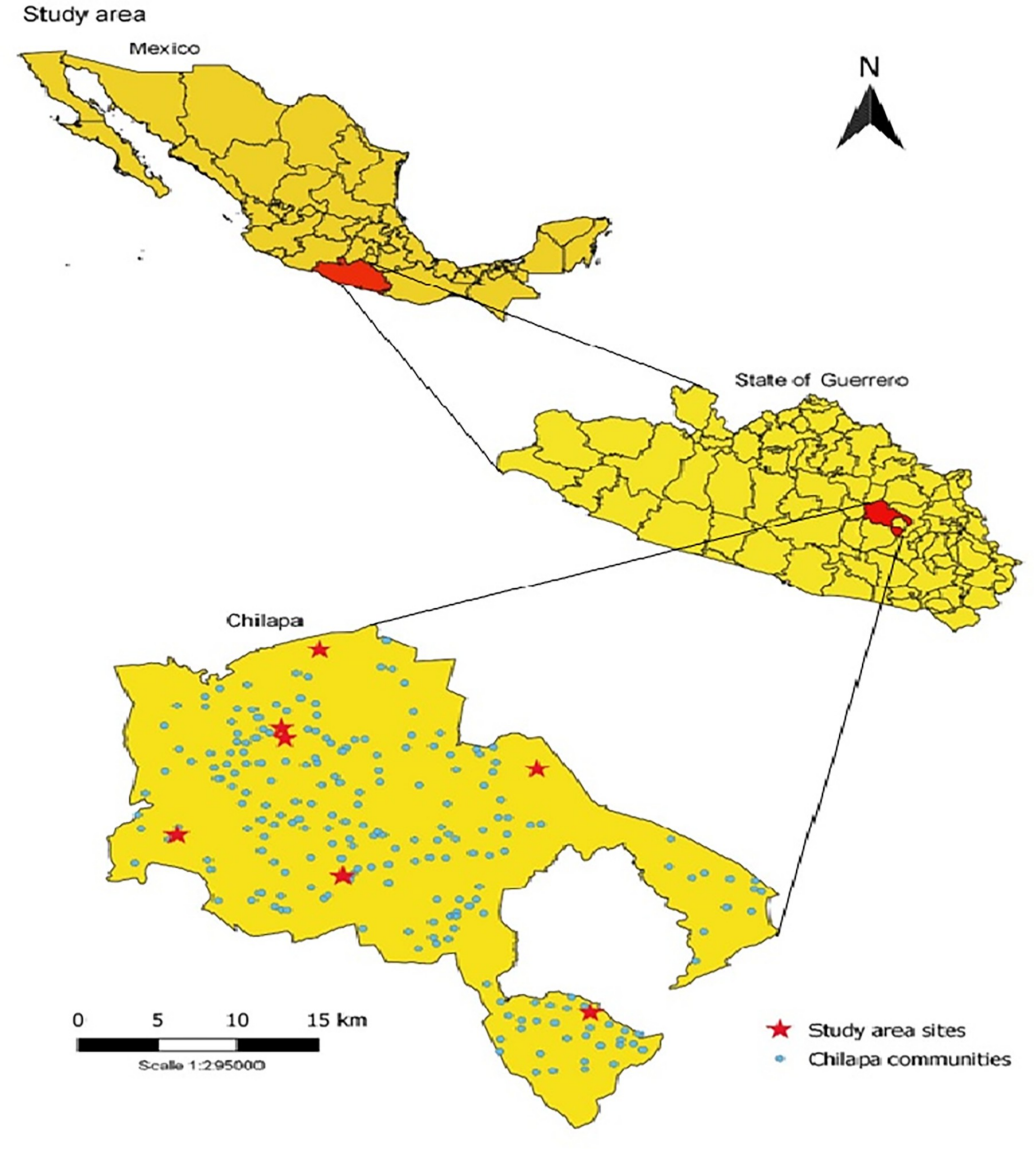
Communities included in the study. Geographical distribution of the communities in the municipality of Chilapa, those marked in red those included in the study. https://www.inegi.org.mx/temas/mg/#Descargas.

This cross-sectional study included seven communities, an average 163 households each, in the municipality of Chilapa, Guerrero, in June 2018 (Fig 1). The purposive sample included four communities to reflect the 58% (655/1144) of rural households and three communities to reflect the 42% (489/1144) of urban households. The survey included all residents in contiguous households in each cluster, independent of their age and gender, with no sub-sampling. We excluded households when no one over the age of 18 years was present to provide the relevant information. We excluded questionnaires that answered less than 80% of the questions.

### Questionnaires and data acquisition

An administered questionnaire collated data about the household including the storage habits for construction material, firewood, ears of corn, and corn stubble; poultry; actions taken to prevent scorpions in the home; and, the language spoken in the home. We recorded for each resident: age; gender; sleeping place; ceiling or mosquito net over the bed; history of scorpion sting in the last year; the location in the home where the sting occurred and the activity carried out at the time; and, helping in the cleaning of the house, food preparation, and handling firewood.

Prior to the survey, a pilot test in a community with similar conditions estimated acceptability, comprehension, and the time to apply the questionnaire. This established the logistics for the fieldwork. We trained six interviewers, who had a minimum of a high school education, to obtain verbal informed consent from the respondents and to administer the questionnaire (S1 Questionnaire).

Before starting fieldwork, we informed the local authorities about the objective of the study and obtained their approval. Members of the community accompanied the interviewers to facilitate access to the households. A sketch map of each community guided the distribution of the interviewers to avoid missing any households. Each interviewer explained the objectives of the study, requested the respondent’s informed consent, and then applied the survey.

### Statistical analysis

We digitized the data with EpiData version 3.1 [25], using double data capture and validation to minimize errors. CIETmap [26] generated simple frequencies with bivariate cluster adjustments as proposed by Lamothe [27]. We estimated the magnitude of the effect using odds ratios (OR), evaluating statistical significance with the Mantel-Haenszel procedure [28], using 95% confidence intervals adjusted cluster (CI95%acl) of Cornfield [29]. Multivariate analysis stepped down from a saturated model including all statistically significant factors in the bivariate analysis, excluded the least significant factor one by one until the final model included only factors significant at the 5% level. We evaluated effect modification using Woolf’s χ^*2*^ heterogeneity test [30].

## Results

The 1,144 households provided information on 4,985 people, of whom 53.5% (2665/4895), were female. Table 1 shows the characteristics of the households surveyed.

**Table 1:** Characteristics of the dwellings surveyed in the communities of Chilapa, Guerrero, Mexico.

Factor		N = 1144	%
Walls of the dwelling	Block	660	57.7
	Cement	260	22.7
	Adobe	119	10.4
	Wood	54	4.7
	Reed	47	4.1
	Cardboard sheeting	4	0.3
Flooring of the dwelling	Cement	970	84.8
	Earth	102	8.9
	Tiled	72	6.3
Roof of the dwelling	Concrete	690	60.3
	Galvanised sheeting	284	24.8
	Asbestos sheeting	82	7.2
	Roof tiles	64	5.6
	Cardboard sheeting, dry palm leaves, reeds	24	2.1

The average age of participants was 29 years (SD±21.1, n = 4985, range 0–96). The overall rate of scorpion stings in the last year was 4.4% (218/4,985), the rate at home being 3% (149/4,985). Some 5.4% (126/2,320) of men received scorpion stings and so did 3.5% (92/2,665) of women, χ^2^ = 63.38, p<0.001.

**Fig 2:**
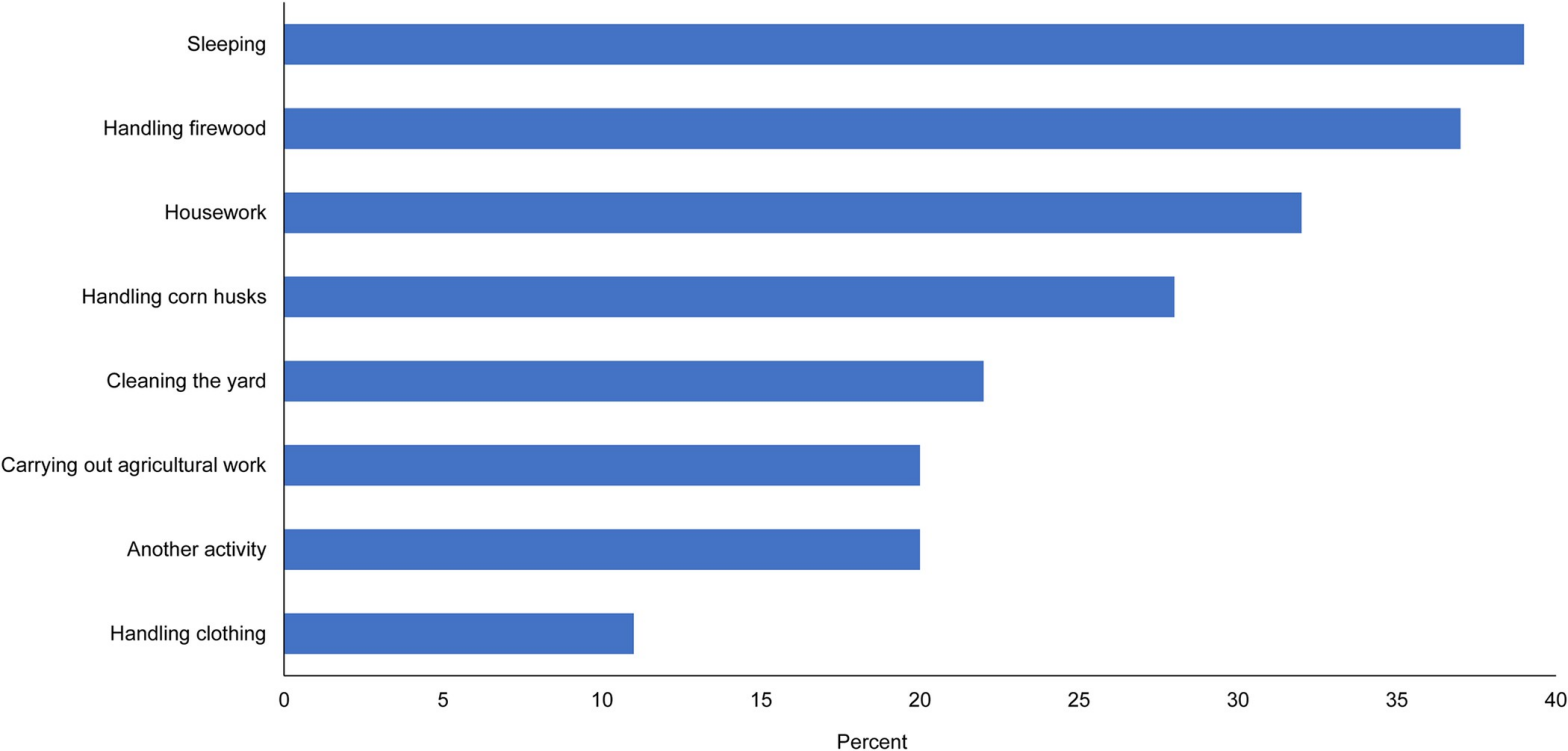
Frequency of scorpion sting by type of activity undertaken at the point that the event occurs, in Chilapa, Guerrero, Mexico.

Of 218 people who reported scorpion stings, 68.3% (149/218) reported the event in or around the dwelling, 26.6% (58/218) in an agricultural environment, 2.8% (6/218) in the street, and 2.3% (5/218) at work. Fig 2 shows the most frequent activities at the moment of the sting. The other activity includes drying with a towel, picking up stones, closing a door, washing hands, reading, handling wires, and planting flowers in a pot.

### Actions to avoid scorpion sting

At a household level, 55.2% (631/1,144) of respondents indicated that they clean their home, 18.6% (213/1,144) fumigate, 14.2% (162/1,144) use domestic insecticides, 6.1% (70/1,144) undertake other preventive actions in the home, and 5.9% (68/1,144) take no actions to avoid scorpion stings. At an individual level, 54.3% (2,707/4,985) indicated having a ceiling or mosquito net over the place where they sleep.

Table 2 shows factors associated with scorpion stings in the bivariate analysis. A person was more likely to be stung by a scorpion if living in a rural area, taking no action to prevent scorpion sting, keeping free-ranging ducks on the patio of the home, keeping hens, storing firewood in the home, storing corn or corn stubble in the home, ≤4 people or less living in the home, being ≥11 years of age, being male, helping with cooking, and carrying firewood.

**Table 2:** Bivariate analysis of factors associated with scorpion sting in the municipality of Chilapa, Guerrero, Mexico.

Factors	Scorpion sting		%	OR	CI95% acl
	Yes	No			
**Area**					
Rural	162	2461	6.2	**2.7**	**1.45–5.08**
Urban	56	2306	2.4		
**Whether a language other than Spanish is spoken in the home**					
Another language	72	1488	4.6	1.1	0.49–2.42
Only Spanish	146	3279	4.3		
**Actions taken to prevent scorpion sting**					
No action taken	40	692	5.5	**1.3**	**1.09–1.60**
Action taken	178	4075	4.2		
**Flooring material in the place where the respondent sleeps**					
Earth	25	383	6.1	1.5	0.88–2.49
Concrete/tiling	193	4384	4.2		
**Wall material in the place where the respondent sleeps**					
Wood or reed	173	3634	4.5	1.2	0.68–2.10
Concrete, breeze block	45	1133	3.8		
**Roofing material in the place where the respondent sleeps**					
Sheeting or palm leaves	91	1769	4.9	1.2	0.79–1.86
Concrete	127	2998	4.0		
**Free-ranging ducks on the patio of the home**					
Yes	28	14	6.8	**1.68**	**1.01–2.80**
No	190	4383	4.2		
**Free-ranging hens on the patio of the home**					
Yes	139	1997	6.5	**2.44**	**1.86–3.20**
No	79	2770	2.8		
**Storing items of little use**					
Yes	132	3013	4.2	0.89	0.64–1.25
No	86	1754	4.7		
**Storing construction material**					
Yes	109	2066	5.0	1.31	0.93–1.83
No	109	2701	3.9		
**Storing firewood in the home**					
Yes	190	3461	5.2	**2.6**	**1.46–4.48**
No	28	1306	2.1		
**Storing corn husks or stubble**					
Yes	71	904	7.3	**2.1**	**1.28–3.33**
No	147	3863	3.7		
**Number of people per household**					
≤4 people	50	798	5.9	**1.5**	**1.04–2.10**
≥5 people	168	3969	4.1		
**Age**					
≥11 years	194	3733	4.9	**2.2**	**1.22–4.10**
≤10 years	24	1034	2.3		
**Gender**					
Man	126	2194	5.4	**1.6**	**1.19–2.18**
Woman	92	2573	3.5		
**Place where the respondent sleeps**					
Floor	13	211	5.8	1.4	0.79–2.37
Bed	205	4556	4.3		
**Wear a mosquito net to sleep**					
No	96	2182	4.2	1.07	0.81–1.43
Yes	122	2585	4.5		
**Helping with cooking**					
Yes	135	2657	4.8	1.3	0.94–1.77
No	83	2110	3.8		
**Helping to carry firewood for cooking**					
Yes	132	1528	8.0	**3.2**	**2.18–4.85**
No	86	3239	2.6		
**Helping with housework**					
Yes	176	3413	4.9	**1.7**	**1.05–2.64**
No	42	1354	3.0		

Table 3 shows the factors associated with scorpion stings in the multivariate analysis. With no test of heterogeneity significant at the 5% level, there was no need to separate the analysis by subgroup. People in this municipality were more likely to be stung if they were carrying firewood, keeping free-ranging hens on the patio of the home, living in a rural area, being male, and helping with housework. The factors exited the saturated model were site; actions taken in the home to avoid scorpion sting; keeping ducks in the home; storing construction material, firewood, and corn husks; the number of people per home, and age.

**Table 3:** Multivariate analysis of factors associated with scorpion sting in the municipality of Chilapa, Guerrero, Mexico.

Factor	ORc	ORacl	CI95%acl
Carrying firewood	3.1	2.1	1.40–3.09
Free-ranging hens in the yard of the home	2.4	1.9	1.19–2.85
Living in a rural area	2.7	1.7	1.04–2.78
Being male	1.6	1.6	1.18–2.28
Doing housework	1.7	1.6	1.04–2.40

## Discussion

One person in every twenty of the study population suffered a scorpion sting in the year leading up to the study, 5.4% of men and 3.5% of women. Risk factors in this setting included carrying firewood, free-ranging hens around the home, living in a rural area, being male, and carrying out housework. The present study aimed to document the occurrence of scorpion stings and associated factors in Chilapa de Alvarez, Mexico.

The occurrence of scorpion stings in Chilapa was similar to the 4.1% found in Colombia by Gómez *et al* [14]. and was lower than the 6 to 15% reported by other studies [17,31]. Villegas *et al* [17] observed a larger gender gap in the same direction as our study, 20.7% for men and 8.7% for women. The lower occurrence of scorpion stings in our study may be because we conducted it at the end of the dry season (June). Higher risk has been observed later in the year [32], with activities like harvesting, transport, and storage of corn increasing the risk of contact with scorpions [32,33].

Most stings occurred while the person was sleeping, reflecting the nocturnal habits of the scorpion. Similar results were reported by Dabo *et al* and Bennett *et al* [34,35]. Scorpions might also seek refuge in beds during the day, staying in beds that are not shaken out until rolled on or otherwise unintentionally bothered by a sleeping person.

We found carrying firewood associated with scorpion stings, supporting the report of Villegas *et al*. [17] who reported handling firewood is the most common activity when stung. Carrying firewood into the home is implicit in the rural use of firewood for cooking [36]. The collector spends more time on the fields and touches the ground more. Because stacking the firewood creates the ideal environment for scorpions, moving a bundle of firewood into the kitchen might dislodge the insect, resulting in an increased risk of stings for those who carry the wood. This could be avoided by restacking wood to be used indoors, piece by piece, instead of grasping a bundle together. Our inability to show an increased risk for those who do the cooking is probably related to the piecemeal addition of wood to the fire. The association with free-range hens is inconsistent with other studies of this factor [16]. A plausible explanation might be scratching by hens moves earth and leaves, causing scorpions to leave their shelters and thus increasing the probability of scorpion sting [37]. Poultry nests made of straw may provide favorable shelters for scorpions and collecting eggs might thus increase the risk of a sting.

**Fig 3:**
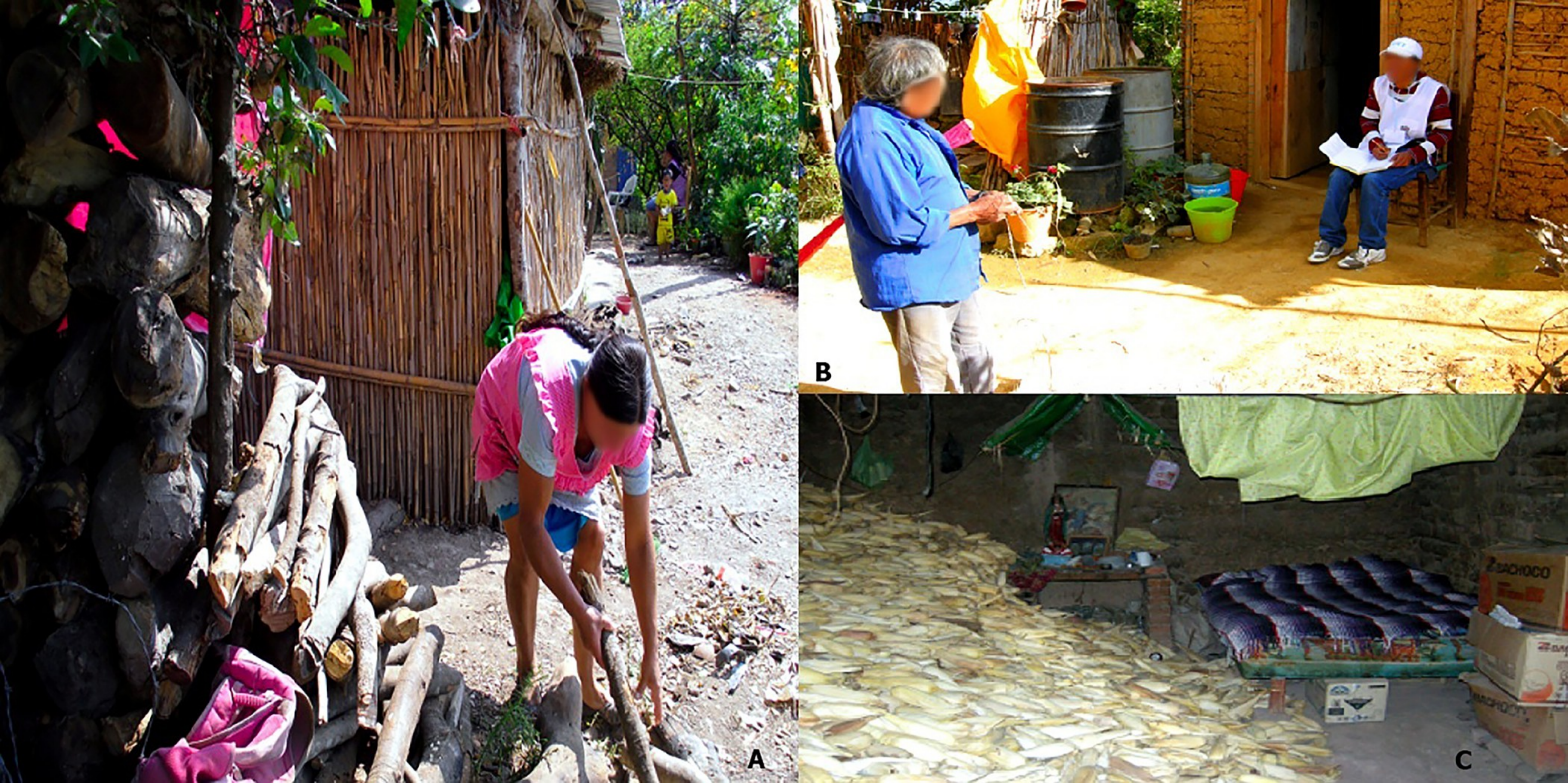
Dwellings provide ideal habitats for scorpion harborage, which can increase the risk of being stung. (A) Storage and handling of firewood for cooking. (B) The walls of unplastered wattle and daub (C) Keeping corn husks inside the house.

Our finding that living in a rural area is a risk factor supports that of Gómez *et al* [14], (OR 2.4; CI95% 1.2–4.7). Rural areas support the presence of scorpions because of homes surrounded by trees and undergrowth [38]. Rural residents also often have to store firewood, corn husks or stubble, work instruments, items of little use, and construction material near their dwellings, offering a habitat for scorpions [39]. Fig 3 illustrates the likely living conditions of scorpions in the study area.

The higher risk for males echoes the finding of Villegas *et al*. [17] in corn-producing communities (OR 1.90; CI95% 1.06–3.40). The association can be explained by men carrying out higher-risk activities like harvesting corn, collecting and transporting firewood, or other types of agricultural work (13,20). Not using protective gloves during the corn harvest increases the risk of scorpion stings [20].

Our finding that helping with housework carries a risk of scorpion sting coincides with the finding of Silva *et al*. [16], although they saw housework in terms of the lack of cleanliness (OR 1.84; CI95% 1.02–4.16). Based on our study, the high-risk actions include moving furniture, kitchen utensils, clothing, and items of little use, which may cause scorpions to leave their shelter and, thus, increase the probability of stings.

The efforts of Mexican health institutions to implement preventive measures have had little preventive impact on these communities. Risks of stings are related to individual activities in the home or in an agricultural environment and environmental conditions. Given the gendered division of work in our area, men are more at risk in agricultural work, while also at risk due to work in the home.

The findings have relevance for populations with similar characteristics to the study area. Chilapa has a warm sub-humid climate, a humidity typical of grasslands or prairies, at an altitude of 1400 meters and above sea level. Although the municipalities have a high diversity and density of scorpions, many of them toxic [40], the evidence-based approach to risk factors and possibly some risk factors found here might be generalizable to other settings.

The results can inform the improvement of prevention and control programs for scorpion stings, allowing local health education programs to draw attention to local risk factors [18,19]. This information could encourage people to take low-cost actions to prevent scorpion stings. These include placing mosquito nets over beds, shaking bed linen before going to bed, and shaking clothes before wearing them, to take account of the nocturnal habits of scorpions. Eliminating weeds around the house would reduce the risk of contact with scorpions that hide in the vicinity. Agricultural workers might be advised to use gloves, especially when picking up wood and harvesting corn, as scorpions hide in the leaves of ripe corn.

### Strengths and weaknesses

We studied a high proportion of households, 95% (1,144/1,205), in the sample communities, with the remaining 61 households opting not to participate. The low rate of refusal was probably due to the community member who accompanied the interviewer, increasing respondent trust in the survey process.

One limitation was that only one family member provided the data on behalf of the entire household. It is possible that the occurrence of scorpion stings observed by the present study may be an underestimation because the respondent may not have known whether one of their family members had undergone such an event. It is also possible that asking about events “in the last year” meant different things to different respondents, making the measured period prevalence less reliable than an inquiry based on a fixed time point, for example, since the beginning of this year, or since last Christmas.

## Conclusion

The study confirms scorpion sting is a neglected public health problem in some marginalized communities. Risk factors include carrying firewood, keeping free-range hens, living in a rural area, being male, and carrying out housework. Strengthening programs for the prevention and control of scorpion stings, via the implementation of low-cost actions at the household, agricultural, and community levels, is necessary to reduce both the number of scorpion stings and their consequences.

## Supporting information

S1 Checklist Strobe checklist. (DOC)

S1 Questionnaire Questionnaire scorpion sting. (DOCX)

S1 Data Dataset from Chilapa. (XLSX)

S2 Data Coding sheet. (XLSX)
