# Supplementary material for: Occurrence of scorpion sting and associated factors in a highly marginalized municipality in Guerrero, Mexico: A cross-sectional study
Source: PLoS Negl Trop Dis. 2023 May 1;17(5):e0011271. doi: 10.1371/journal.pntd.0011271 (PMC10150966; doi:10.1371/journal.pntd.0011271)
Supplement: S1 Questionnaire — (DOCX) [file pntd.0011271.s002.docx]

Questionnaire

Occurrence of scorpion sting and associated factors in the municipality of Chilapa de Álvarez, Guerrero, Mexico: a cross-sectional study

What other language do you speak at home besides Spanish?

What do you do at home to avoid being stung by scorpions?

How long have you been doing this?

What material is the floor where you sleep made of?

What material are the walls where you sleep?

What about the ceiling where you sleep?

What do you do in the house where you are most in danger of being stung by a scorpion?

Do you or your neighbor's ducks run loose in your backyard?

Do you or your neighbor's chickens run loose in your yard?

Do you usually keep utilities inside or outside your house?

Do you usually keep construction materials inside or outside the house?

Do you usually keep firewood inside or outside the house?

Do you usually keep corn husks or stubble inside or outside the house?

How many people live in this house?

Can you tell me the ages of the men, starting with the youngest?

Can you tell me the ages of the women, starting with the youngest?

The one who is...years old

What do you sleep in?

Does he/she use a sky or canopy above where he/she sleeps?

Does this same person help with cooking?

Does he/she help carry firewood for cooking?

Does this person help with the cleaning of the house?

Have you been stung by a scorpion this year?

In what month were you stung?

What were you doing when you were stung?

Where were you when you were stung by the scorpion?
